# Nonessential amino acids in tropical ruminant feed: Investigating grass and legume forages of Indonesia

**DOI:** 10.5455/javar.2023.j739

**Published:** 2023-12-31

**Authors:** Gresy Eva Tresia, Yenny Nur Anggraeny, Wiwik Heny Winarsih, Setiasih Setiasih, Eni Siti Rohaeni, Mariyono Mariyono, Dicky Pamungkas

**Affiliations:** 1 Research Center for Animal Husbandry, Research Organization for Agriculture and Food, National Research and Innovation Agency Cibinong Science Center, Cibinong Science Center, Cibinong, Indonesia; 2 East Java Regional Research and Innovation Agency, Surabaya, East Java, Indonesia

**Keywords:** Cattle, concentrate, grass, legume, metabolism

## Abstract

**Objective:**

This study aimed to examine the concentration of nonessential amino acids (NEAAs) in ruminant feed in tropical areas, with a focus on forage grasses and legumes in Indonesia.

**Materials and Methods:**

A total of 11 grasses (*Chrysopogon zizanioides, Brachiaria brizantha, Brachiaria humidicola, Paspalum dilatatum, Paspalum atatum, Chloris gayana, Pennisetum polystachion, Panicum maximum, Cenchrus biflorus, Andropogon canaliculatus,* and *Digotaria decumbens*) and six legumes (*Arachis hypogaea, Pueararia Javanica, Centrosema pubescens, Clitoria ternatea,* and *Arachis pintoi*) were analyzed for NEAA content using high-performance liquid chromatography (HPLC).

**Results:**

Based on the results of this research, it was found that almost all NEAA content in forage was less than 3% (Serine (Ser), Alanine (Ala), Glycine (Gly), Tyrosine (Tyr), Proline (Pro), Cysteine (Cys), and Asparagine (Asn), except for glutamic (Glu) acid and arginine (Arg) in some legumes. The Glu content in grass ranges from 0.76% to 2.61%, and the Arg content ranges from 0.92% to 2.09%. These two NEAAs were most abundant in grasses and legumes, with concentrations of 5.10% to 6.27% and 3.10% to 5.53%, respectively.

**Conclusion:**

Our study concluded that Glu and Arg were the most abundant NEAAs in tropical forages in Indonesia, with legumes having a higher concentration of NEAAs compared to grasses. Among the legumes, *A. hypogaea* had the highest NEAA content (23.40%), while among the forages, *C. zizanioides* had the highest NEAA content (12.37%). However, it was observed that neither legumes nor grasses could fulfill the metabolizable TNEAAs requirements for gaining cattle (250 kg of empty body weight gain), unlike commercial concentrates, which were found to meet the requirements, especially for Arg, Glu, and thyronine. The provision of concentrate is necessary to supplement forage to meet the NEAA needs for cattle.

## Introduction

Several studies that have been published in recent years show that protein for livestock has gone beyond the use of crude protein feed and is currently more focused on meeting the needs of ammonia and AA from the rumen fermentation process for microbial protein synthesis and AA needs. Amino acids (AAs) are considered important for the survival, growth, and conception development of ruminant livestock [1]. AAs are mainly used for the synthesis of proteins and other nitrogen compounds of physiological importance. AAs are divided into categories of essential and non-essential nutrients; it is found that the term “NEAA” is considered a misnomer in nutritional science [2,3]. Research reveals the importance of NEAA. Certain NEAAs function in gene expression in animal cells, micro-RNA biogenesis, epigenetics, and T-cell activation and function [4,5], and most NEAAs participate in cell signaling through kinases to regulate nutrient metabolism [2]. Several studies have reported that NEAA plays a role in influencing the digestive and absorption functions of the small intestine [2,6].

It has been assumed that NEAA can be synthesized by animal and human bodies to meet the needs for maximum growth and optimal health [7]. Several studies have been conducted reporting that NEAA supplementation in feed can improve production performance in pigs, poultry, and fish [8–15], as well as in cattle. Lean et al. [16] reported in a meta-analysis study that NEAA had a positive production response to milk yield and milk protein. Early lactating cows cannot achieve maximum milk protein production without a post-ruminal supply of Gln or Pro [17,18]. Wu et al. [19] reported that L-Gln supplementation in calf diets could ameliorate the adverse effects of abrupt weaning. Likewise, administration of glutamine (Gln) to Holstein dairy cows can improve total antioxidant status and plasma glutathione peroxidase activity [20]. This opinion firmly states that NEAA is conditionally indispensable for mammalian metabolic needs and mammalian survival. Furthermore, it is known that the rate of NEAA synthesis is influenced by the availability of essential AA (EAA) and glucose, species, race, age, physiological status, and disease [2].

Despite the assumption that NEAAs can be synthesized sufficiently by animals and humans, studies have shown that supplementing NEAAs in the diet could improve production performance in various livestock species. However, there was limited information available on the AA composition of tropical forages commonly used in ruminant feed in Indonesia. Therefore, this study aimed to assess the concentration of NEAAs in grass and legume forages in Indonesia to provide valuable information for formulating diets for ruminants.

## Materials and Methods

### Ethical approval

This research did not involve the use of animals.

### Feed samples and preparation

The samples used and selected for this research were grass and legumes, considering that they represent a variety of tropical forages commonly given to animals (Table 1). Grass leaves (11 types) and legumes (6 types) were collected from the experimental field of the Indonesian Beef Cattle Research Institute, Pasuruan, East Java. These leaves were dried in an oven at 60°C, and ground with a hammer mill (screen size 1 mm). The concentrate used in this research was concentrate for dairy cattle (KSA) and concentrate for beef cattle (KST). The reason for selecting grass and legume samples for this research was based on the extent of their cultivation and use by farmers in animal husbandry. The samples of grasses and legumes in this study are justified because of their widespread cultivation and use in animal husbandry. These grasses and legumes are known to be very suitable and adapted to the agricultural environment, so they are in great demand by farmers. In addition, they are very popular with livestock. Grasses and nuts were carefully collected at the pre-bloom stage, which is considered optimal for processing as research samples.

### AA analysis

High-Performance Liquid Chromatography (HPLC) was used for determining the nonessential AA (NEAA) content in the samples, following the AOAC Official Method 994.12 [21] for sample hydrolysis and determination. Ten milliliters of 6N hydrochloric acid containing 0.1% phenol were used to dilute the sample, which was subsequently hydrolyzed under reflux at 110°C for 24 h. After cooling each hydrolysate sample to room temperature, 20 ml of the norleucine standard solution was added using a volumetric pipette. The hydrolysates, filtered through a sintered glass filter, were placed in 1,000-ml evaporating flasks. After removing the flask from the evaporator, 50 ml of sodium citrate buffer were added and transferred to a 50-ml polyethylene bottle. The filtrate from the hydrolysate samples, filtered through a sintered glass filter into a 250-ml vacuum flask, was transferred to a 250-ml beaker. Neutralization of the hydrolysates was performed by adding 40 ml of 7.5 NaOH and adjusting the pH to 2.2 with 2M NaOH. Using a diluted aliquot of the hydrolysate and sodium citrate buffer for sampling, the pH was adjusted to 2 using 2M NaOH. The aliquots containing neutralized hydrolysate samples were diluted with water. Subsequently, after passing through a 0.2-mm filter, 20 ml of the filtrate underwent HPLC analysis.

### Chemical score

The chemical score was calculated using the modified equation by Bouba et al. [22], with the formula as follows:

Chemical score (%)= gm test protein mg of the same nonessential  amino acid in requirement pattern ×100

The metabolizable TNEAA requirements for a gain of cattle (250 kg empty body weight gain) by Amaral et al. [23] were used for this purpose: aspartic (Asp) acid (25.83/100 gm protein), Ser (12.89/100 gm protein), Glu (19.15/100 gm protein), Pro (16.44/100 gm protein), Gly (16.85/100 gm protein), Ala (19.75/100 gm protein), thyronine (10.76/100 gm protein), Arg (12.80/100 gm protein), and cysteine (Cys) (5.54/100 gm protein).

**Table 1.** Forages and concentrates used in the analysis of the amino acids profile.

Item	Species/types	Common name
Grass	*Andropogon canaliculatus*	Andropogon
	*Brachiaria brizantha*	Bread grass
	*Cenchrus ciliaris*	Buffel grass
	*Digitaria decumbens*	Pangola grass
	*C. zizanioides*	Vertiver
	*Pennisetum polystachion*	Thailand/Mission grass
	*Chloris gayana*	Rhodes grass
	*Brachiaria humidicola*	Koronivia grass
	*Panicum maximum*	Guinea grass
	*Paspalum dilatatum*	Dallis grass
	*Paspalum atratum*	Atratum grass
Legume	*Centrosema pubescens*	Centro
	*Arachis hypogaea*	Groundnut
	*C. ternatea*	Butterfly pea
	*Pueraria triloba*	Kudzu vein
	*Arachis pintoi*	Pinto peanut
	*Pueraria javanica*	Kudzu bean
Concentrates	Dairy cattle concentrates (local product)	KSA
	Beef cattle concentrates (local product)	KST

### Data analysis

The descriptive analysis of the AA content in grass and legume forage, as well as concentrate, was conducted using the mean and standard error of the mean.

## Results

### NEAA composition

The proportion of NEAAs in grass and legume forage, as well as concentrate, is presented in Table 2. The results showed that legumes (six species) have a higher percentage of total NEAAs (TNEAA) and each NEAA at about 14.34%–24.56%, compared to grasses (11 species), which have a lower percentage of about 2.76%–12.34%. However, it is confirmed that both KSA and KST concentrates have higher concentrations of NEAA than forages. Among the grasses, *Chrysopogon zizanioides* had the highest TNEAA concentration, while *Digitaria decumbens* had the lowest. Among the legumes, *Pueraria triloba* had the highest TNEAA concentration, while *Clitoria ternatea* had the lowest.

The content of AAs in grasses followed a trend. Glu > Arg > Ser > Ala > Gly > Tyr > Pro > Cys > Asn. The content of AAs in legumes followed a trend. Glu > Arg > Ala > Tyr > Gly > Ser > Pro > Cys > Asn. The content of AAs in concentrate followed a trend, i.e., Glu > Arg > Ala > Gly > Tyr > Ser > Pro > Cys > Asn. In the present study, the major components of NEAA in the concentrate group were Glu and Arg, which contained more than 20%, and Asn and Cys, which contained less than 5%. Similarities also occurred in the grass and legume groups. However, in the grasses group, the major component of NEAA ranged between 0.76% and 2.61% for Glu and 0.58% and 2.10% for Arg, but the minor component of NEAA ranged between 0.08% and 0.2% for Asn and 0.17% for Cys. The vertiver grass (*C. zizanioides*) has a high content of Ser, which was an outlier among the grass groups, following Arg and Glu. The major component of NEAA in the legume group ranged between 3.75% and 6.18% for Glu and 3.10% and 5.02% for Arg, but the minor component of NEAA was between 0.26% and 0.39% for Asn and 0.31% and 0.50% for Cys.

**Figure 1.**
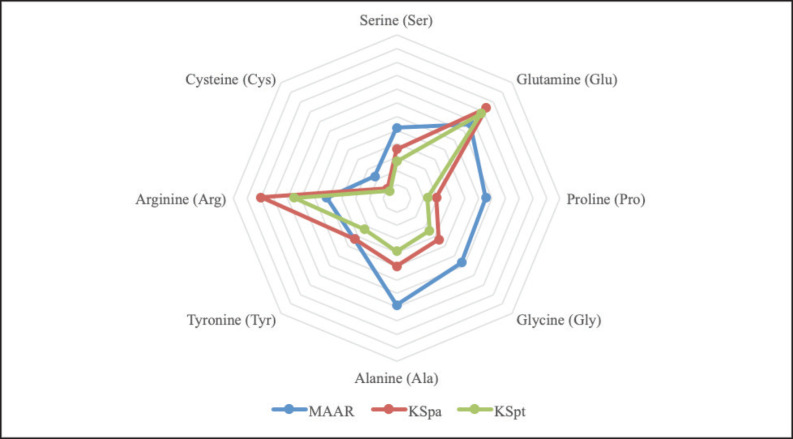
Nonessential amino acids of concentrate.

**Figure 2.**
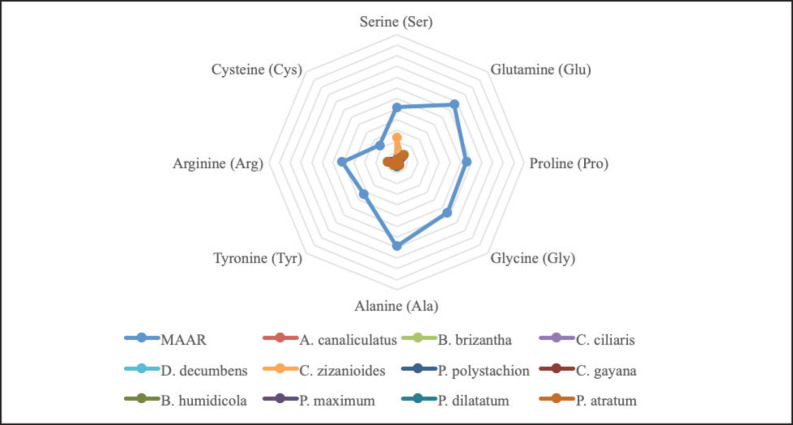
Nonessential amino acids of grasses.

**Figure 3.**
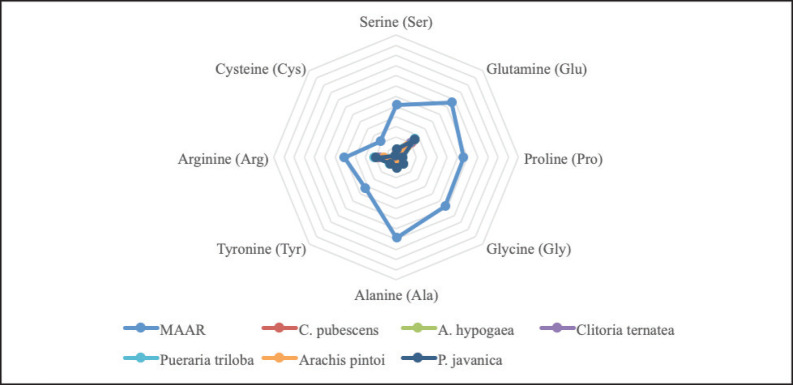
Nonessential amino acids of legumes.

The results presented in Figure 1 indicate that both legumes and grasses are insufficient to provide the metabolizable TNEAA requirements for weight gain in cattle weighing 250 kg. On the other hand, commercial concentrates were found to meet these requirements, especially for Arg, Glu, and thyronine. However, it is worth noting that grasses have a high Ser content (Fig. 2) and legumes tend to have a high Arg and Glu content (Fig. 3), even though they do not fully meet the NEAA metabolism requirements.

### Ratio of essential and NEAA composition

Table 3 demonstrates that all of the feed samples had an average EAA/NEAA ratio greater than 1, except for vertiver grass. The EAA/NEAA ratio for grasses ranged from 0.64 to 1.34, and for legumes, it ranged from 1.11 to 1.16. Meanwhile, the EAA/NEAA ratio for concentrates was approximately 1.13–1.19.

### Chemical score of NEAA composition

The chemical score of NEAAs in grass and legume forages is presented in Table 4. Neither the grasses nor legumes could provide at least 50% of the metabolizable NEAA requirements for gaining cattle with a 250 kg empty body weight gain. The legumes could provide a maximum of 39.22% (Arg) of the requirement. The Glu and Arg components of both types of concentrates were able to fulfill the metabolizable NEAA requirements for gaining cattle with a 250 kg empty body weight gain. There was a possibility that KSA concentrate could meet the requirement of metabolizable tyrosine (Tyr). These results showed that concentrates could meet half of the metabolizable Ser, Gly, and alanine (Ala).

## Discussion

New approaches for determining AA requirements in poultry and pigs have been recently described [2,^3^,^10^,14]. However, our understanding of the precise AA needs of ruminants remains limited. Moreover, the practical assessment of feed to determine the ideal balance of essential and non-essential AAs crucial for optimal growth, reproduction, lactation, and maintenance is yet to be established. Identification of the ideal protein profile will provide thorough insights into the protein quality of individual feed components and entire diets, elucidating the specific contribution of each AA in attaining the optimal pattern.

**Table 2.** Nonessential amino acid composition (gm/100 gm protein) of three groups of ruminants feed sources.

Feedstuffs	Nonessential amino acid (gm/100 gm of CP)								
	Asn	Ser	Glu	Pro	Gly	Ala	Tyr	Arg	Cys
**Concentrat**e									
KSA	1.85	8.90	23.32	7.31	10.96	12.54	10.76	24.91	2.34
KST	1.45	6.72	21.94	5.71	8.52	9.80	8.23	18.82	1.78
Mean	1.65	7.81	22.63	6.51	9.74	11.17	9.49	21.86	2.06
SEM	0.20	1.09	0.69	0.8	1.22	1.37	1.26	3.05	0.28
**Grasses**									
*A. canaliculatu*s	0.08	0.38	1.17	0.26	0.40	0.48	0.40	0.92	0.09
*B. brizanth*a	0.17	0.77	2.61	0.62	0.91	1.10	0.91	2.09	0.19
*C. ciliari*s	0.10	0.48	1.43	0.33	0.43	0.59	0.52	1.21	0.14
*D. decumben*s	0.05	0.26	0.76	0.16	0.26	0.30	0.25	0.58	0.06
*C. zizanioide*s	0.12	5.83	1.88	0.44	0.67	0.78	0.67	1.55	0.17
*P. polystachion*	0.10	0.46	1.57	0.36	0.55	0.65	0.54	1.24	0.14
*C. Gayana*	0.11	0.58	1.62	0.37	0.58	0.67	0.57	1.28	0.16
*B. humidicola*	0.15	0.70	2.38	0.56	0.86	1.00	0.87	2.00	0.18
*P. maximum*	0.10	0.49	1.45	0.33	0.52	0.6	0.54	1.26	0.14
*P. dilatatum*	0.14	0.69	2.21	0.52	0.80	0.93	0.62	1.99	0.19
*P. atratum*	0.15	0.75	2.28	0.54	0.83	0.10	0.91	2.09	0.20
Mean	0.11	1.04	1.76	0.41	0.62	0.65	0.62	1.47	0.15
SEM	0.01	0.48	0.17	0.04	0.06	0.09	0.06	0.15	0.01
**Legumes**									
*C. pubescens*	0.34	1.58	5.10	1.25	1.87	2.17	1.97	4.53	0.40
*A. hypogaea*	0.38	1.87	6.18	1.52	2.25	2.63	2.19	5.02	0.45
*C. ternatea*	0.32	1.55	5.01	1.22	1.86	2.17	1.81	4.15	0.43
*Pueraria triloba*	0.40	1.90	6.27	1.54	2.31	2.67	2.42	5.53	0.50
*A. pintoi*	0.26	1.12	3.75	0.91	1.38	1.59	1.35	3.10	0.31
*P. javanica*	0.38	1.87	6.18	1.52	2.25	2.63	2.19	5.02	0.45
Mean	0.35	1.65	5.41	1.33	1.99	2.31	1.99	4.56	0.42
SEM	0.02	0.12	0.41	0.10	0.15	0.17	0.15	0.35	0.03

Sources of feed for ruminants consist of forages, both grasses and legumes, and supplementation from concentrates. Our study showed the concentrates contain NEAAs and are more complete than others because they are a mixture of several ingredients. The concentrate for dairy cows (KSA) and beef cattle (KST) consists of rice bran, cassava meal, wheat pollard, soy sauce waste, copra meal, palm kernel meal, and coffee husk; however, the proportion of these feedstuffs was different in the formulation of KSA and KST. The formulation of dairy cow concentrate (KSA) in this study had a greater TNEAA content than the concentrate in the study by Zhao et al. [25], which was 8.97%–9.3% of DM (calculated NEAA and Arg). Luo et al. [26] reported a range of 4.17%–23.22% for TNEAA content in grains. Moreover, Li et al. [27] reported that the range of TNEE in plant-based and animal-source feedstuffs was 4.62–37.51 and 23.86%–81.99%, respectively. This suggests that both types of feedstuffs (grains and animal sources) can be utilized as forage supplements, effectively addressing the need to balance their AA content and deliver all of the necessary functional AAs.

Glutamic (Glu) acid was most abundant in all studied feedstuffs, whereas Asn had the lowest essential AA content. The same observation was made on 11 animal-source feedstuffs and three plant-source feedstuffs (spirulina meal, soybean meal, and soy protein concentrate) [28]. Of the two source feedstuffs, they reported that the animal source feedstuff was more abundant in free NEAA content. In addition, the highest AA content in animal sources is Glu (190–14,578 mg/kg as fed basis), Ala (343–16,955 mg/kg as fed basis), and thyronine (1,180–12,467 mg/kg as fed basis), while in plant sources it is Glu (106–6,232 mg/kg as fed basis), Asp (88.7–605 mg/kg as fed basis), and Arg (174–2,097 mg/kg as fed basis) [28]. According to Li et al. [27], it was shown that the majority of feed ingredients from plants have low levels of cyanide (less than 0.25%), but relatively high amounts of glutamic acid and Gln (15%–20%), as well as branched chain AAs (15%–25%). Nonetheless, a significant portion of plant-based animal feed consists of limited proportions of overall AAs [27]. Likewise, a review by Watford [29] stated that glutamate and Glu are widely present AAs found in most types of food, making up approximately 5% to 15% of dietary protein.

According to Kumar et al. [30], there was a common pattern among the various plants in terms of their AA composition, with glutamic acid being the most prevalent, followed by aspartic acid, Arg, Leucine (Leu), Ala, Valine (Val), Pro, Ser, Threonine (Thr), Gly, Phenylalanine (Phe), Isoleucine (IIe), Tyr, Asn, Gln, Histidine (His), Cys, Methionine (Met), and Tryptophan (Trp) in descending order of abundance. This finding indicates that plant, environmental condition, stress, and metabolism pathways affect free AA composition in legumes and grasses. For instance, Yang et al. [31] demonstrated that regulation of lysine metabolism differed between plant species. Additionally, Capstaff and Miller [32] explained that stresses and defoliation can change the nutritional content, including AAs, in forage crops. Moreover, a study by Edmunds et al. [33] showed different AA content in forages due to differences in harvest age, maturity, and conservation type.

**Table 3.** Ratio of essential and nonessential amino acids of three groups of ruminants feed sources.

Feedstuffs TAA		Total amino acids (g/100 g of CP)			EAA/NEAA
		EAA*	NEAA		
Concentrate	KSA	226.16	123.28	102.88	1.20
	KST	176.66	93.71	82.96	1.13
	Mean	201.41	108.49	92.92	1.16
	SEM	24.75	14.79	9.96	0.03
Grasses	*A. canaliculatus*	8.85	4.67	4.18	1.12
	*B. brizantha*	20.02	10.66	9.36	1.14
	*C. ciliaris*	11.38	6.15	5.23	1.18
	*D. decumbens*	5.62	2.96	2.66	1.11
	*C. zizanioides*	19.81	7.71	12.10	0.64
	*P. polystachion*	11.68	6.08	5.60	1.09
	*C. Gayana*	12.49	6.55	5.94	1.10
	*B. humidicola*	18.64	9.93	8.71	1.14
	*P. maximum*	11.68	6.28	5.41	1.16
	*P. dilatatum*	18.88	10.79	8.09	1.34
	*P. atratum*	18.07	10.25	7.83	1.31
	Mean	14.28	7.46	6.83	1.12
	SEM	1.50	0.79	0.81	0.05
Legumes	*C. pubescens*	41.30	22.08	19.21	1.15
	*A. hypogaea*	47.83	25.35	22.48	1.13
	*C. ternatea*	38.87	20.35	18.52	1.10
	*Pueraria triloba*	50.91	27.36	23.55	1.16
	*A. pintoi*	29.50	15.73	13.77	1.14
	*P. javanica*	47.37	24.89	22.48	1.11
	Mean	42.63	22.63	20.00	1.13
	SEM	3.20	1.71	1.49	0.01

*Sources: Pamungkas et al. unpublished [24]

**Table 4.** Chemical score (%) of three groups of ruminants feed sources.

Feedstuffs	Chemical score based on metabolizable amino acid requirements (%)								
	Aspara-Asn)	Serine (Ser)	Glutamine (Glu)	Proline (Pro)	Glycine (Gly)	Alanine (Ala)	Thyronine (Tyr)	Arginine (Arg)	Cysteine (Cys)
**Concentrate**									
KSA	7.16	69.03	121.77	44.43	65.02	63.51	99.98	194.63	42.22
KST	5.60	52.10	114.58	34.71	50.58	49.64	76.48	146.99	32.08
Mean	6.38	60.56	118.17	39.57	49.30	56.57	88.2	149.27	21.53
SEM	0.77	8.46	3.59	4.86	6.05	6.93	11.75	20.89	2.92
**Grasses**									
*A. canaliculatus*	0.29	2.94	6.12	1.59	2.40	2.43	3.68	7.17	1.66
*B. brizantha*	0.65	5.98	13.60	3.78	5.38	5.54	8.45	16.34	3.45
*C. ciliaris*	0.38	3.74	7.45	1.98	2.58	2.98	4.87	9.48	2.51
*D. decumbens*	0.20	1.99	3.94	0.95	1.54	1.52	2.28	4.50	1.16
*C. zizanioides*	0.46	45.23	9.79	2.66	3.98	3.95	6.23	12.09	2.98
*P. polystachion*	0.38	3.58	8.17	2.19	3.28	3.29	4.98	9.70	2.45
*C. gayana*	0.41	4.48	8.45	2.27	3.46	3.40	5.25	10.03	2.82
*B. humidicola*	0.58	5.45	12.43	3.43	5.13	5.06	8.05	15.59	3.32
*P. maximum*	0.38	3.79	7.55	2.01	3.06	3.02	5.05	9.81	2.47
*P. dilatatum*	0.55	5.33	11.55	3.18	4.74	4.69	5.77	15.52	3.45
*P. atratum*	0.57	5.78	11.91	3.28	4.90	0.48	8.42	16.30	3.61
Mean	0.44	8.02	9.17	2.48	3.15	3.30	5.72	10.25	1.25
SEM	0.04	3.73	0.89	0.26	0.32	0.45	0.59	1.05	0.15
**Legumes**									
*C. pubescens*	1.33	12.23	26.65	7.59	11.10	10.98	18.35	35.37	7.26
*A. hypogaea*	1.48	14.50	32.25	9.23	13.34	13.32	20.35	39.22	8.12
*C. ternatea*	1.25	11.99	26.14	7.44	11.04	10.98	16.80	32.41	7.80
*Pueraria triloba*	1.56	14.72	32.75	9.37	13.73	13.52	22.44	43.23	9.01
*A. pintoi*	0.99	8.71	19.58	5.52	8.17	8.04	12.56	24.23	5.67
*P. javanica*	1.48	14.51	32.25	9.23	13.34	13.31	20.35	39.22	8.10
Mean	1.34	12.77	28.27	8.06	10.09	11.69	18.48	31.27	4.34
SEM	0.08	0.95	2.12	0.62	0.77	0.88	1.42	2.38	0.35

It has been stated that fulfilling the required AA intake is essential for the health and existence of both animals and humans [1,^8^,^10^,11]. However, as shown in studies with mice [34], elevated levels of dietary non-essential AAs, which are excessive, can have a detrimental effect on health and may even impact lifespan. Furthermore, according to Romano et al. [34], this leads to the concept of ‘optimal nutrition’ being difficult to define due to various factors such as dietary goals, sex, age, health, environment, and species. Previously described in a review of the dietary essentiality of nutritionally NEAAs for animals and humans [2], Wu et al. [19] observed that dietary Gln supplementation may increase lamb immunological function by supplying energy. Indeed, this finding was also emphasized by Ma and Ma [35]. Brake and Swanson [36]explained that small intestinal starch digestion (SISD) is increased by greater postruminal flows of individual AA (i.e., Glu). Single feedstuffs or their mixture containing large amounts of rumen-undegradable protein and Glu can be used to increase ruminant development, lactation, and pregnancy, particularly in heat-stressed lactating cows [27]. For each gram of duodenal Glu flow, small-intestine starch digestion improved by 0.96–1.25 gm/day [37,38]. Meanwhile, Herring et al. [39] mentioned that Arg supplementation in pregnant ewes promotes embryonic survival since Arg is essential for the survival, growth, and development of the conceptus. Wu et al. [40,41] explained that: (1) feed for pigs must contain adequate Arg and Gln to optimal normal fetal, neonatal, and post-weaning growth; (2) feed for early cows must contain enough Pro, glutamate, and Gly to support maximum growth and feed efficiency; and (3) feed for lactating sows must contain sufficient Arg and glutamate for optimal milk production. Choi and Coloff [42] state that Glu and glutamate (NEAA) have a role in overcoming cancer in humans; this shows that NEAA has an important role in cancer pathology. Manipulation of food in NEAA metabolism is one strategy that can potentially inhibit tumor growth. Strategies for manipulating NEAA in food need to be carefully developed to avoid negative side effects. Despite the fact that the importance of non-essential AAs (NEAAs) has been recognized and incorporated in current feed studies, the ratio of EAAs to NEAAs needs to be considered.

Several studies have also demonstrated that giving an adequate combination of essential and NEAAs promotes an increase in body weight, which is regarded as a suitable metric to assess the animal’s success in terms of growth and wellness [41,43]. Romano et al. [34] reported strengthening the effects on mice’s lifespan of varying diets that contained different proteins or free EAA/NEAA ratios but the same amount of carbohydrates, lipids, micronutrients, and nitrogen. The highest EAA diet ratio of EAA/NEAA approximately 100/0) was found to extend lifespan, maintain a low body weight, lower systemic inflammation, and maintain a balanced protein metabolism. However, a higher NEAA intake (the ratio of EAA to NEAA is approximately 0/100) is associated with a shorter lifespan and worse health. According to Corsetti et al. [44], there was a decrease in body weight, body length, and organ weight with high dietary NEAA (EAA/NEAA ratio 33/67 and 0/100). The authors proposed supplementing EAA to enhance the EAA/NEAA ratios to at least 1 [34]. Interestingly, our result pointed out that the feedstuffs (concentrate, legume, and grasses) had an EAA/NEAA ratio >1 excluding vertiver grass (*C. zizanioides*). Meanwhile, a study by Abd El-Gawad et al. [45] found the ratio EAA/NEAA value in sunflower meal, soybean meal, and cotton seed meal was 0.91, 0.89, and 0.81, respectively. Dietary intake of these source proteins in buffalo resulted in an EAA/NEAA ratio in milk of 0.99–1.09.

This NEAA chemical calculation was modified from the calculation of the chemical score of essential AAs carried out by Bouba et al. [22]. The authors reported that the chemical score is a useful tool for evaluating proteins that contain essential AAs that are not synthesized by the human body. The mixture of feedstuffs (concentrate) indicated that concentrate, besides providing a good proportion of EAA and NEAA, also meets the metabolizable NEAA requirements for cattle gain (250 kg of empty body weight gain) reported by Bouba et al. [22]. Numerous nutritional, physiological, pathological, and environmental factors influence the amount of NEAA required in the diet [46]. Even though the current study found that legumes and grasses did not meet the requirement, that does not mean livestock should be given NEAA supplementation. In addition, according to previous authors, lysine, Met, and His are most commonly identified as the most limiting AAs, and rumen-protected forms of lysine and Met are available for supplementation [47].

Apart from technical problems, there are large gaps in our understanding of some fields. The effects of ideal patterns of essential and NEAAs for optimal growth, reproduction, lactation, and maintenance need further investigation given the significance of the nutritional value of AAs. In order to achieve this, analyses of the relationships between the degradable protein in these three feedstuff groups and the rumen’s AA profile, as well as correlations with NDF digestibility, rumen microbial biomass production, and the rumen microbe population, are required.

## Conclusion

Glu and Arg were the most abundant in all studied feedstuffs. Legumes had a higher concentration of NEAAs compared to grasses. From the six legumes analysis, all of them had higher NEAA concentrations compared to 11 tropical grasses in Indonesia, with *A. hypogaea* legume having the highest concentration of NEAA (23.40%), while *C. zizanioides* had the highest NEAA concentration (12.37%) among the forages. However, the study showed that neither legumes nor grasses could provide the required metabolizable TNEAAs for weight gain in cattle (250 kg of empty body weight gain). Therefore, a balanced diet that meets the essential and NEAA requirements of cattle is crucial for optimal growth and weight gain. Commercial concentrates can be a useful supplement to forage-based diets to meet NEAA requirements in cattle. The weakness of this study is the lack of a large number of samples analyzed, and the analysis of samples can be differentiated based on different agroecosystems specific to plants. This weakness also serves as a suggestion for further research that can be tried on livestock by taking into account essential and non-essential AAs.

## List of abbreviations

AA (s), amino acid(s); NEAA(s), nonessential amino acid(s); EAA(s), essential amino acid(s); Glu, Glutamic acid; Asn, Asparagine; Arg, Arginine; Leu, Leucine; Ala, Alanine; Val, Valine; Pro, Proline; Ser, Serine; Thr, Threonine; Gly, Glycine; Phe, Phenylalanine; IIe, Isoleucine; Tyr, Tyrosine; His, Histidine; Cys, Cysteine; Met, Methionine; Trp, Tryptophan; Asp, Aspartic acid; HPLC, High- performance liquid chromatography; KSA, concentrate for dairy cattle; KST, concentrate for beef cattle; NDF, Neutral Detergent Fiber.
